# Formulation Development and Stability Studies of Norfloxacin Extended-Release Matrix Tablets

**DOI:** 10.1155/2013/716736

**Published:** 2013-09-08

**Authors:** Paulo Renato Oliveira, Cassiana Mendes, Lilian Klein, Maximiliano da Silva Sangoi, Larissa Sakis Bernardi, Marcos Antônio Segatto Silva

**Affiliations:** ^1^Post Graduation Program in Pharmaceutical Sciences, Universidade Estadual do Centro Oeste (UNICENTRO), 85040-080 Guarapuava, PR, Brazil; ^2^Post Graduation Program in Pharmacy, Health Science Centre, Federal University of Santa Catarina, Quality Control Laboratory, J/K 207, 88040-900 Florianópolis, SC, Brazil; ^3^Laboratory of Quality Control and Pharmaceutical Analysis, Faculty of Pharmacy, Federal University of Rio de Janeiro, 27930-560 Macaé, RJ, Brazil

## Abstract

The aim of this research was to develop a new hydrophilic matrix system containing norfloxacin (NFX). Extended-release tablets are usually intended for once-a-day administration with benefits to the patient and lower discontinuation of the therapy. Formulations were developed with hydroxypropylmethylcellulose or poly(ethylene oxide) as hydrophilic polymers, with different molecular weights (MWs) and concentrations (20 and 30%). The tablets were found to be stable (6 months at 40 ± 2°C and 75 ± 5% relative humidity), and the film-coating process is recommended to avoid NFX photodegradation. The dissolution profiles demonstrated an extended-release of NFX for all developed formulations. Dissolution curves analyzed using the Korsmeyer exponential equation showed that drug release was controlled by both drug diffusion and polymer relaxation or erosion mechanisms. A more erosion controlled system was obtained for the formulations containing lower MW and amount of polymer. With the increase in both MW and amount of polymer in the formulation, the gel layer became stronger, and the dissolution was more drug-diffusion dependent. Formulations containing intermediate MW polymers or high concentration (30%) of low MW polymers demonstrated a combination of extended and complete in vitro drug release. This way, these formulations could provide an increased bioavailability in vivo.

## 1. Introduction

Hydrophilic matrix tablets are among the most popular orally administered controlled release systems. Despite having been around since four decades, matrices are still the reference starting point for innovations in drug delivery. It can be due to the fact that they are considered quite reliable in terms of drug delivery, simple technology, and low cost of manufacture. Moreover, matrices that can be continuously innovated as new materials for formulation became commercially available [1–5].

The matrix tablets are usually composed of active pharmaceutical ingredients (APIs) and hydrophilic swellable polymers. When the system is exposed to the aqueous medium, water will be absorbed and a gel layer will be formed. This viscous gel layer may hinder water penetration and become the rate-controlling step during gel formation. The gel strength is important in the matrix performance and is dependent on the chemical structure, concentration, and viscosity of the polymer used. Depending on the mechanical properties of the gel layer, drug release is controlled by different mechanisms and kinetics. Polymer swelling, drug dissolution, drug diffusion, and matrix erosion are the basic phenomena leading to the drug release from swellable matrices [6–12]. Additionally, drug load and solubility can influence the release mechanism and kinetics.

Hydroxypropylmethylcellulose (HPMC) is a propylene glycol ether of methylcellulose and is widely used as a matrix former in oral controlled release tablet formulations [1]. One of its most important characteristics is the high swellability, which has a significant effect on the release kinetics of an incorporated drug. Furthermore, HPMC is compatible with numerous drugs, accommodates high levels of drug loading, and can be easily incorporated to form matrix tablets by direct compression or granulation [9, 13–16]. The availability of a wide range of viscosity grades also allows the formulator to modify the release of drugs from HPMC matrix tablets according to therapeutic need.

High molecular weight poly(ethylene oxides) (PEOs) have been proposed as an alternative to HPMC in controlled release dosage forms [17]. They are important polymers for the pharmaceutical industries mainly because of their nontoxicity, high water solubility and swellability, insensitivity to the pH of the biological medium, and ease of production. PEOs swell and form a compact gel layer on the surface of the tablet which is responsible for the controlled drug release [17–22]. They are also available in a wide range of molecular weights, thus allowing the formulator to control the mechanism of drug release to achieve the therapeutic goal. 

Norfloxacin (NFX) is a synthetic broad spectrum antibacterial drug being the firstly selected drug for the treatment of diseases caused by *Campylobacter*, *E. coli*, *Salmonella*, *Shigella*, and* V. cholera* [23, 24]. The drug is also used for the treatment of urinary tract infections as well as gonorrhoea and infection of eyes [23]. The recommended dosage is usually 400 mg twice daily. The half-life of NFX in serum and plasma is 3-4 hours and only approximately 30–40% of an oral dose is absorbed [25, 26]. Increasing bacterial resistance to currently available antibiotics, including the quinolone class, has reduced their effectiveness, making the therapeutic decisions more difficult, and may compromise future use of this class of drugs [27–31]. 

The development of an extended-release formulation that could improve the bioavailability of NFX and reduce the administration schedule may improve the patients' comfort and compliance, resulting in lower discontinuation of the therapy, with consequently a decrease in bacterial resistance. The correct choice of the hydrophilic polymer, molecular weight, and quantity in the matrix formulation can provide an appropriate combination of polymer swelling, erosion, or drug diffusion mechanisms to control drug release. Thus, the aim of this work was to develop and carry out stability and in vitro dissolution studies of a new formulation of norfloxacin extended-release tablets.

## 2. Materials and Methods

### 2.1. Materials

Norfloxacin (NFX) was purchased from Zhejiang Neo-Dankong Pharmaceutical (Zhejiang, China). Hydroxypropylmethylcellulose (HPMC) K100LV (apparent viscosity: 100 mPa s, 2% in water at 20°C), HPMC K4M (4000 mPa s), poly(ethylene oxide) (PEO) N60K (2000 kDa), and PEO 301 (4000 kDa) were kindly donated by Colorcon (São Paulo, Brazil). The pharmaceutical excipients used were microcrystalline cellulose (Microcel 102, Blanver, Itapevi, Brazil), magnesium stearate (M. Cassab, São Paulo, Brazil), and colloidal silicon dioxide (Labsynth, Diadema, Brazil). 

### 2.2. Methods

#### 2.2.1. Preparation of Matrix Tablets

A powder blend containing NFX, polymer, and microcrystalline cellulose was prepared and mixed for 15 min, followed by addition of magnesium stearate and colloidal silicon dioxide with a further 5 min mixing. The modules having the composition reported in Table 1 were prepared by direct compression using a 19 × 8 mm punch set (Fellc compressing model F-10/8, São Paulo, Brazil).

#### 2.2.2. Characterization of Tablet Formulation

Tablets were characterized by weight, hardness, friability, dimension, and loss on drying according to pharmacopeial limits [32, 33]. The average weight was obtained for at least 20 units. Hardness was determined for at least 10 tablets using a Hardness Tester (298-AT, Nova Ética, Vargem Grande Paulista, Brazil) and adopting a minimum hardness of 3 kgf as the acceptance criterion. For each formula, friability was evaluated for a sample of 20 tablets, using the acceptance criterion of a maximum loss of 1.5% of the initial weight. Dimension was evaluated measuring 10 tablets with a paquimeter. Loss on drying was carried out with 2 g of sample, in vacuum, at 105°C for 2 h.

#### 2.2.3. Tablet Coating and Blistering

A tablet coating solution was formed by adding 30 g of Opadry II White (Colorcon, São Paulo, Brazil) to 120 g of purified water and stirring for 2 min. An amount corresponding to 50% of each formulation batch was placed in a Rama Cota RD conventional coating machine. Tablets were preheated until the bed temperature reached 45°C. Pan rotation was set to 40 rpm, and tablets were coated using a Binks Model 460 spray gun operating at a pressure of 2 bar. The coating solution was pumped at a rate of 5.9–9.6 g/min using a peristaltic pump. Tablet bed temperature was maintained between 42 and 45°C during the spray coating process. After coating, an amount of coated and uncoated tables were blistered in transparent PVC blister and sealed with an aluminium foil.

#### 2.2.4. NFX Tablets Assay

NFX quantification assay was carried out according to a previously validated method [34]. Briefly, the LC system was operated isocratically at 40°C using a mobile phase composed by phosphoric acid 0.04 M, pH 3.0/acetonitrile (84 : 16; v/v), eluted at a flow rate of 1.0 mL/min. A reversed-phase Phenomenex (Torrance, USA) Luna C_18_ column (150 mm × 4.6 mm I.D., with a particle size of 5 *μ*m and pore size of 100 Å) was used, and the detector was set at 272 nm. The injection volume was 20 *μ*L. 

To prepare the sample stock solution, the manufactured extended-release tablets were crushed to a fine powder. An appropriated amount was transferred into an individual 50 mL volumetric flask, dissolved with 0.2 mL of glacial acetic acid, and diluted to volume with mobile phase, obtaining a concentration of 1 mg/mL of the API. The NFX standard stock solutions were prepared by weighing 50 mg, transferred to 50 mL volumetric flasks, dissolved with 0.2 mL of acetic acid glacial, and diluted to volume with mobile phase, obtaining a concentration of 1 mg/mL. Both sample and standard stock solutions were stored at 2–8°C protected from light. Working solutions were prepared daily by diluting the stock solutions to an appropriate concentration in mobile phase.

#### 2.2.5. Stability Tests

The manufactured tablets were submitted to accelerated stability test. Samples of each batch (non-coated, coated, with and without blister) were maintained for 6 months in an accelerated stability chamber (420 CLD, Nova Ética, Vargem Grande Paulista, Brazil) at 40 ± 2°C and 75 ± 5% relative humidity [35, 36]. For photostability tests, samples were exposed to an overall illumination of not less than 1.2 million lux [37]. The illumination was measured with a Digital Lux Meter (MLM-1011, Minipa, São Paulo, Brazil). Protected samples (wrapped in aluminium foil) were used as dark controls to evaluate the contribution of thermally induced change to the total observed change. 

#### 2.2.6. Drug Release Study

Drug release studies were performed based on pharmacopeial methods using USP apparatus II Vankel 7000 dissolution tester (Varian Technology Group, Cary, USA), with paddle rotation of 75 rpm, in 900 mL of buffer pH 4.0 at 37.0 ± 0.5°C [32, 33]. At specified time intervals, 5 mL samples were withdrawn, filtered, and quantified in a UV spectrophotometer (Varian Cary 50 bio, Cary, USA) at the wavelength 278 nm.

#### 2.2.7. Analysis of Drug Release

The analysis of the values obtained in dissolution tests is easier when mathematical formulas that express the dissolution results as a function of some of the dosage forms characteristics are used. NFX release kinetic was evaluated according to the following models: zero order, first order, Higuchi, and Korsmeyer-Peppas. Additionally, the difference factor (*f*1) and similarity factor (*f*2) were used to compare the dissolution profiles.

*Zero-Order Model.* Drug dissolution from pharmaceutical dosage forms that do not disaggregate and release the drug slowly (assuming that area does not change and no equilibrium conditions are obtained) following a “steady-state release” can be represented by (1) [38]:

(1) $$\begin{array}{c} Q_{t}=Q_{0}+k_{0}t, \end{array}$$

where *Q*_*t*_ is the fraction of drug released at time *t*; *Q*_0_ is the initial amount of drug in the solution (most times *Q*_0_ = 0);  *k*_0_ is the zero-order release constant. The pharmaceutical dosage forms following this profile release the same amount of drug by unit of time, and it is the ideal method of drug release in order to achieve a pharmacological prolonged action.

*First-Order Model.* The drug dissolution is assumed to decline exponentially, and the release rate is proportional to the residual amount of drug in the dosage form (2) [38]:

(2) $$\begin{array}{c} \log Q_{t}=\log Q_{0}+\frac{k_{1}t}{2.303}, \end{array}$$

where *Q*_*t*_ is the fraction of drug released at time *t*; *Q*_0_ is the initial amount of drug in the solution;  *k*_1_ is the first-order release constant. The pharmaceutical dosage forms following this dissolution profile release the drug by unit of time in a way that is proportional to the amount of drug remaining in its interior. 

*Higuchi Model.* It is the most widely used model to describe drug release from matrices, which is derived from Higuchi for a planar matrix. It describes the drug release mechanism as a diffusion process based on Fick's law, dependent on the square root of time (3) [38, 39]:

(3) $$\begin{array}{c} Q_{t}=K_{H}\sqrt{t}, \end{array}$$

where *Q*_*t*_ is the fraction of drug released at time  *t*  and  *K*_*H*_  is the Higuchi dissolution constant. 

*Korsmeyer-Peppas Model. *This model is generally used to analyze the release of pharmaceutical polymeric dosage forms when the release mechanism is not well known or when more than one type of release phenomena could be involved (4) [38, 40]:

(4) $$\begin{array}{c} \frac{M_{t}}{M_{\infty }}=kt^{n}, \end{array}$$

where *M*_*t*_/*M*_*∞*_ is the fraction of drug released, *k* is the kinetic constants characteristic of the drug/polymer, *n* is the diffusional exponent for drug release. Dissolution values in the range of 5–60% were used to fit release data.

*Difference Factor *(*f*1)* and Similarity Factor *(*f*2). The relevance of the difference between the release curves were assessed using difference factor *f*1 and similarity factor *f*2, calculated by (5) and (6), respectively [41, 42]:

(5) $$\begin{array}{c} f1=\left\{\frac{\sum_{i=1}^{n}\left(R_{t}-T_{t}\right)}{\sum_{i=1}^{n}R_{t}}\right\}\cdot 100, \end{array}$$

(6) $$\begin{array}{c} f2=50\cdot \log\left\{{\left[1+\left(\frac{1}{n}\right)\sum_{i=1}^{n}{\left(R_{t}-T_{t}\right)}^{2}\right]}^{-0.5}\cdot 100\right\}, \end{array}$$

where *R*_*t*_ and *T*_*t*_ are the percentages released at each time point.

An *f*1 value up to 15 (0–15) and *f*2 value between 50 and 100 implies similarity between two release profiles. Only one more point after the 85% of drug has been released was used for the equation.

## 3. Results and Discussion

Norfloxacin matrix tables were successfully obtained by direct compression. Different polymers and molecular weights did not interfere in the technological process. The pharmacopeial characteristics of the manufactured tablets are summarized in Table 2. These results demonstrated that the tablets were reliable on hardness and friability, which are important characteristics for the further step of coating. 

Consistent hardness of the tablet surface enables the coating to “lock” into the surface. If the surface is too soft, the impingement of the solution can erode the tablet. Too hard a surface will not allow the solution to impinge and adhere, and the coating will peel away. Both of these coating defects can also occur by over- or underapplying the coating solution or by applying the coating with too much or too little force [43–46]. The film-coating (Opadry II) applied on the NFX tablets surface is nonfunctional; however, it can improve the final quality by protecting the hygroscopic polymer from absorbing humidity and preventing photodegradation of the drug. NFX coated tablets showed a uniform, smooth, and shiny surface, without coating defects. From Table 2, it can be observed that the weight and hardness increased about 3% and 9%, respectively, demonstrating the influence of the coating process. The loss on drying analysis (Table 2) showed that the coated tablets have a lower amount of volatile matter, probably due to the loss of water absorbed during the coating process at 42–45°C.

The assay determination of NFX demonstrated that all formulations were in the range from 99.43 to 102.35% (Table 3). Therefore, the coating process did not influence the assay of the drug. 

Accelerated stability testing was carried out to provide evidence of how the quality of the manufactured tablets may change with time under the influence of environmental factors such as temperature and humidity. Brazil, being considered with hot and humid climate, is classified in region IV [35]. According to this classification, the accelerated stability study was carried out for 6 months in a climatic chamber at 40 ± 2°C and 75 ± 5% relative humidity. The obtained results are shown in Table 3. All formulations were considered stable since after 6 months a change from the initial assay of 5% or more was not observed [35]. The presence of coating and/or blister did not influence the stability of the developed tablets. Additionally, the chromatographic profiles did not show any additional degradation peak. 

**Figure 1:**
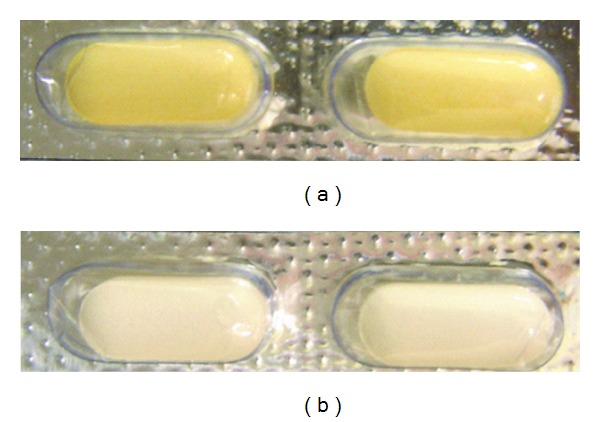
Norfloxacin blistered matrix tablets after photostability study: uncoated (a) and coated (b).

Light testing should be an integral part of stress testing and recommends evaluation of the photostability of a formulation to demonstrate that light exposure does not result in unacceptable changes [35, 37]. For this study, the following formulations were selected: HPMC K100LV (20 and 30%) and PEO N60K (20 and 30%). At the end of the exposure period (about five days), equivalent of not less than 1.2 million lux, samples were examined for changes in appearance and for assay. A color change from pale-yellow to dark-yellow in NFX raw material and uncoated tablets was observed. The transparent blister (primary packing) did not have any protecting influence on the formulations (Figure 1). Prolonged exposure of NFX bulk drug, tablets, and specially in solution under direct sunlight or fluorescent light results in the formation of ethylenediamine degradation product [47, 48]. Since the chromatograms did not show additional peaks and a significant decrease of drug content was not observed (assays between 98.17 and 100.80%), it seems that the ethylenediamine degradant requires an exposure time and/or intensity higher than the used in this research to be significantly formed. Nonetheless, to prevent drug exposure to light and degradation, the coating process or light-protective blister for the formulations would be recommended. 

Two concentrations (20 and 30%) of different MWs HPMC or PEO polymers were used to manufacture the NFX matrix tablets used in this study (Table 1). The dissolution test was carried out under sink conditions, defined as the volume of medium being at least three times higher than that necessary to obtain a saturated solution of the drug [32]. Samples were withdrawn from the dissolution medium at the following times: 0.5, 1, 2, 3, 4, 5, 6, 7, 8, 9, 10, 11, 12, and 24 h. The first time point at 0.5 h was included to study if the product presents a burst effect (with an excessive early drug release), while the final time point shows whether or not the intended dose is fully delivered. NFX is an amphoteric drug with minimal solubility in water at pH between 4.0 and 10.0. This way, the dissolution studies were carried out in buffer pH 4.0 as described in the U.S. Pharmacopoeia (glacial acetic acid and sodium hydroxide) for NFX tablets [32]. In addition, further information could be obtained during the formulation development step by carrying out dissolution studies in gastric and intestinal simulated fluids.

**Figure 2:**
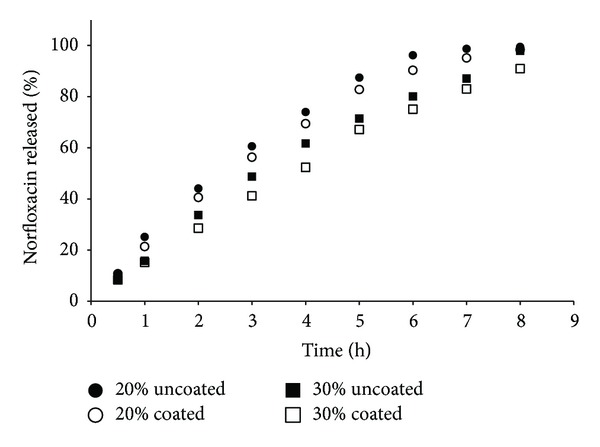
Norfloxacin released versus time of matrix tablets containing HPMC K100LV.

**Figure 3:**
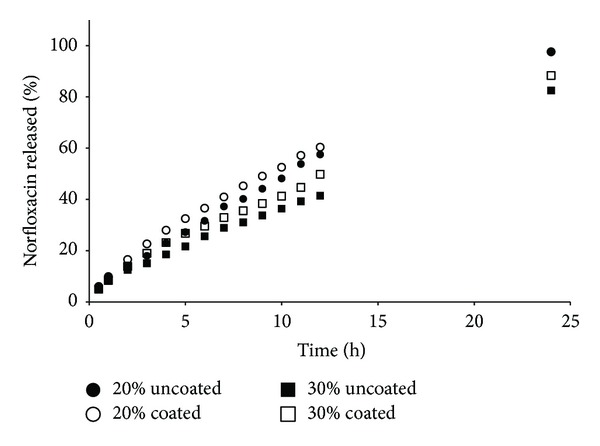
Norfloxacin released versus time of matrix tablets containing HPMC K4M.

**Figure 4:**
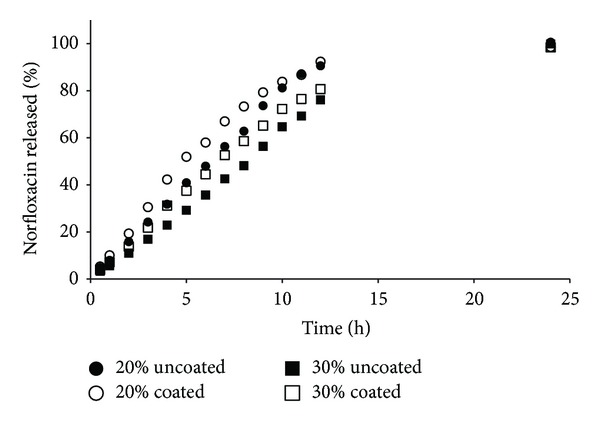
Norfloxacin released versus time of matrix tablets containing PEO N60K.

**Figure 5:**
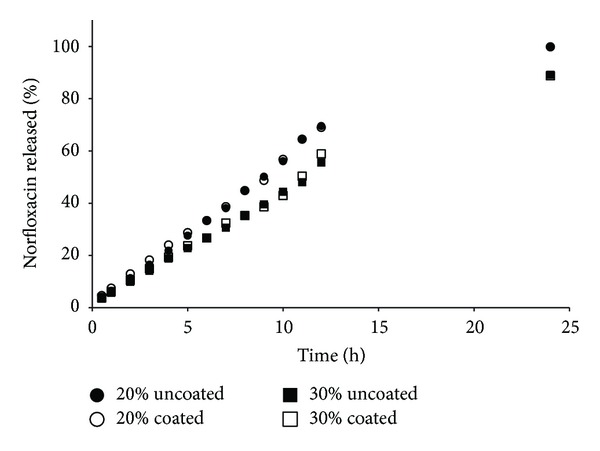
Norfloxacin released versus time of matrix tablets containing PEO 301.

NFX release profiles are shown in Figures 2–5. The polymers used have different average MWs, and therefore they differ in controlling drug release from matrix tablets. An extended-release of NFX was obtained for all formulations manufactured, demonstrating that the mechanical strength of the viscous-gel layer was strong enough to maintain its integrity and drug release. Faster dissolution was obtained for formulations containing lower MW polymer and concentration (20%) (Figures 2 and 4). The tablets containing HPMC K100LV showed the fast dissolution profile, with complete drug release at about 6–8 h (Figure 2). 

For the formulations containing HPMC K4M (Figure 3) and PEO 301 (Figure 5), the NFX release could be considered complete only at 24 h. Due to the high MW and/or concentration of polymer in the formulations, the swelling was slow and the gel strength was very high, resulting the central part of the tablet not being fully wetted or hydrated (a “dry core”), with slow drug release. It was particularly notable for the formulations containing 30% of these polymers.

It seems that the coating process somehow influenced the NFX dissolution profile (Figures 2–5), and a relation with the polymer MW could be suggested. In general, coated formulations exhibited faster drug release than uncoated ones. The faster NFX release may be due to the coating process temperature that resulted in lower residual humidity tablets (Table 2) and consequently a faster water uptake and polymer swelling in the dissolution medium. 

For HPMC K100LV formulations, due to the lower MW, water uptake, polymer hydration, and gelification is faster than dissolution of the coating film. In this case, the coating may have worked as a “barrier,” and drug release was delayed. For PEO 301 the dissolution profiles were overlapped, demonstrating no influence of the coating. It can be explained since high MW polymers form a stronger gel layer, with lower water uptake rate and drug release, hence influencing drug diffusion and dynamics of matrix erosion. However, the influence of the coating process was not relevant based on the difference (*f*1) and similarity (*f*2) parameters calculated (Table 4).

Dissolution profiles were analyzed for zero-order, first-order, and Higuchi models with the equations up to 12 h of drug release, except for HPMC K100LV 20 and 30% formulations where the equations were analyzed for up to 6 and 8 h, respectively. The analysis according to Korsmeyer-Peppas was carried out with the diffusional exponential equation up to 60% of drug released [40]. Calculation of the exponent *n* identifies the prevalent mechanism of release. For cylindrical systems, *n* = 0.45 indicates diffusion-controlled (Fickian) drug release, and *n* = 0.89 indicates swelling/erosion-controlled drug release (case-II transport). Values of *n* between 0.45 and 0.89 can be regarded as an indicator for the superposition of both phenomena, indicating that the drug delivery was not controlled only by diffusion but also by significant polymer relaxation or erosion mechanisms (anomalous transport). The *n* > 0.89 values reveal a super case-II transport. This mechanism could result from an increased plasticization at the relaxing boundary (gel layer) and is also related to polymer relaxation and erosion mechanisms [40, 49, 50].

In general, data of all matrices provided better fit to Korsmeyer-Peppas model (Tables 5 and 6). No formulation fitted to Higuchi equation, demonstrating that NFX release mechanism was not a diffusion process dependent on the square root of time.

The formulations containing PEO demonstrated also a good fit to zero-order kinetics. The exponent *n* calculated (Tables 5 and 6) for Korsmeyer-Peppas equation confirmed this to PEO N60K (*n* between 0.94 and 1.0) and to PEO 301 (*n* about 0.87), indicating super case-II and case-II transport mechanism, respectively, as also evidenced by quasi-linear release profiles (Figures 4 and 5). HPMC K100LV formulations demonstrated a similar release profile to PEO N60K, where a super case-II transport mechanism was obtained due to the dissolution of polymeric matrix and relaxation of the polymer chain, with zero-order release. 

Based on the dissolution profiles, HPMC K100LV 30%, HPMC K4M 20%, PEO N60K 20%, and PEO N60K 30% matrices presented a combination of polymer type, MW, concentration, and complete drug release that could result in a formulation able to resist to the destructive forces within the gastrointestinal tract, providing a superior in vivo performance. In fact, the results obtained confirm that gels showing lower strength and texture, usually derived from low MW polymers, have lower resistance to the fluid erosion action and the release of the active molecule is mainly due to polymer relaxation and chains disentanglement, leading to drug delivery kinetic towards an erosion/relaxation mechanism, with exponent *n* ≥ 0.89. On the other hand, when the MW or polymer concentration is increasing, the gel layer formed will be concomitantly characterized by higher strength and consistence, being less susceptible to erosion and chains disentanglement, with drug release mechanism tending to diffusion (with decreasing exponent *n* values). 

For quinolones, the activity is partly related to the ratio between the serum peak concentration and the minimum inhibitory concentration of the offending organism [24, 51]. This way, together with in vitro dissolution analysis, in vivo bioavailability studies are critical to obtain a formulation with the desired pharmacokinetic and toxicity profiles. A successful example of a commercially available quinolone extended-release dosage forms is ciprofloxacin (1000 mg) extended-release tablets. Compared to the immediate-release (500 mg, twice-daily administration), the ER formulation provided higher maximum plasma concentrations with lower interpatient variability, with the therapeutic drug levels being achieved rapidly and maintained over the course of 24 h, with good tolerability, and safety [52–54]. After a complete formulation development, the final extended-release NFX dosage form could be a convenient, well-tolerated and effective therapy mainly for urinary tract infections that may improve patients' compliance with treatment and thus decrease the risk of treatment failure and the spread of antibiotic resistance, being an alternative to the commercially available ciprofloxacin extended-release tablets.

## 4. Conclusions

In this study, the development of a stable extended-release dosage form containing norfloxacin was demonstrated. The film-coating of tablets was necessary to avoid a photo-induced color changing of the active pharmaceutical ingredient. The dissolution studies showed that according to the increase in polymer molecular weight and concentration, the matrix changed from a more erodible system (with zero-order release) to a system with dissolution controlled by drug diffusion and polymer relaxation/erosion mechanisms. The formulations containing intermediate molecular weight HPMC or PEO or high concentration (30%) of low molecular weight polymers (HPMC K100LV 30%, HPMC K4M 20%, PEO N60K 20%, and PEO N60K 30%) are more promising, since a combination between gel structure and complete in vitro drug release was obtained. This prolonged and complete in vitro release profile is expected to lead to an increased bioavailability; however, in vivo studies are necessary to confirm this possibility. Based on an improved bioavailability combined with a reduced frequency of administration, an improved patient compliance and decreased bacterial resistance could be achieved. 

## Figures and Tables

**Table 1:** Composition of tablets containing hydroxypropylmethyl cellulose (HPMC) or poly(ethylene oxide) (PEO).

Composition	For one tablet	For one tablet
Norfloxacin	700 mg	700 mg
Polymer	20%	30%
HPMC K100LV		
HPMC K4M		
PEO N60K		
PEO WSR 301		
Magnesium stearate	1%	1%
Colloidal silicon dioxide	0.5%	0.5%
Microcrystalline cellulose	q.s.	q.s.

Total weight	1.07 g	1.07 g

**Table 2:** Pharmacopeial characteristics of norfloxacin matrix tablets.

Formulation	Weight (g)^a^		Hardness (KgF)^b^		Water loss (%)		Friability (%)^a^
	Uncoated	Coated	Uncoated	Coated	Uncoated	Coated	Uncoated
HPMC K100LV 20%	1.1154	1.1472	16.3	17.0	7.75	7.29	0.021
HPMC K100LV 30%	1.1025	1.1321	14.8	16.4	7.93	7.60	0.014
HPMC K4M 20%	1.1033	1.1314	13.1	15.1	7.08	6.60	0.034
HPMC K4M 30%	1.0795	1.1119	13.0	14.5	7.29	7.11	0.018
PEO N60K 20%	1.0858	1.1193	14.2	15.7	7.02	5.08	0.017
PEO N60K 30%	1.0854	1.1131	16.9	17.7	6.77	5.64	0.021
PEO 301 20%	1.0831	1.1162	16.6	18.8	7.16	6.91	0.018
PEO 301 30%	1.1014	1.1306	16.0	17.5	7.09	7.01	0.023

^a^Mean of twenty determinations; ^b^mean of ten determinations.

**Table 3:** Assay results of accelerated stability test.

Formulation	Time zero		After 6 months			
			Blister		Without blister	
	Uncoated (%)	Coated (%)	Uncoated (%)	Coated (%)	Uncoated (%)	Coated (%)
HPMC K100LV 20%	101.98	101.09	101.16	101.51	102.03	101.18
HPMC K100LV 30%	101.47	101.81	101.87	103.01	100.87	101.74
HPMC K4M 20%	101.45	100.42	100.44	102.98	101.92	101.08
HPMC K4M 30%	100.88	99.31	99.12	99.45	99.91	98.09
PEO N60K 20%	102.06	101.72	102.89	98.47	101.44	100.26
PEO N60K 30%	99.43	99.92	98.49	99.05	99.44	99.03
PEO 301 20%	99.48	99.35	101.24	97.51	101.75	100.31
PEO 301 30%	99.71	100.50	99.03	100.49	100.61	100.36

**Table 4:** Difference factor (*f*1) and similarity factor (*f*2) calculated for uncoated and coated norfloxacin matrix tablets.

Formulation	f1	f2
HPMC K100LV 20%	5.47	71.72
HPMC K100LV 30%	8.50	62.32
HPMC K4M 20%	9.22	70.03
HPMC K4M 30%	14.23	66.36
PEO N60K 20%	13.77	56.35
PEO N60K 30%	14.66	57.81
PEO 301 20%	2.00	91.85
PEO 301 30%	3.12	88.95

**Table 5:** Coefficients of determination (*r* ^2^) obtained from dissolution of norfloxacin uncoated formulations according to different mathematical models.

Formulation	Zero order	First order	Higuchi	Korsmeyer-Peppas	
	*r* ^2^	*r* ^2^	*r* ^2^	*r* ^2^	*n*
HPMC K100LV 20%	0.9794	0.9247	0.9685	0.9952	0.9623
HPMC K100LV 30%	0.9794	0.8538	0.9681	0.9985	0.9761
HPMC K4M 20%	0.9943	0.9916	0.9604	0.9963	0.7115
HPMC K4M 30%	0.9834	0.9954	0.9801	0.9986	0.6593
PEO N60K 20%	0.9976	0.9302	0.9372	0.9994	0.9485
PEO N60K 30%	0.9978	0.9513	0.9127	0.9990	1.0027
PEO 301 20%	0.9978	0.9591	0.9180	0.9978	0.8900
PEO 301 30%	0.9978	0.9795	0.9308	0.9971	0.8771

**Table 6:** Coefficients of determination (*r* ^2^) obtained from dissolution of norfloxacin coated formulations according to different mathematical models.

Formulation	Zero order	First order	Higuchi	Korsmeyer-Peppas	
	*r* ^2^	*r* ^2^	*r* ^2^	*r* ^2^	*n*
HPMC K100LV 20%	0.9827	0.9719	0.9649	0.9990	0.9283
HPMC K100LV 30%	0.9898	0.9571	0.9588	0.9999	0.8867
HPMC K4M 20%	0.9833	0.9981	0.9807	0.9998	0.7270
HPMC K4M 30%	0.9784	0.9921	0.9821	0.9984	0.7041
PEO N60K 20%	0.9759	0.9716	0.9685	0.9975	0.9892
PEO N60K 30%	0.9955	0.9766	0.9468	0.9992	1.0019
PEO 301 20%	0.9970	0.9593	0.9277	0.9979	0.8522
PEO 301 30%	0.9911	0.9586	0.9216	0.9917	0.8550
